# Multiparametric MRI before and after Focal Therapy for Prostate Cancer: Pearls and Pitfalls for the Reporting Radiologist

**DOI:** 10.1148/rycan.240269

**Published:** 2025-02-21

**Authors:** Anna L. Lai, Jyothirmayi Velaga, Kae Jack Tay, Guanqi Hang, Yu Guang Tan, John S. P. Yuen, Christopher W. S. Cheng, Nye Thane Ngo, Yan Mee Law

**Affiliations:** ^1^Department of Diagnostic Radiology, Singapore General Hospital, Outram Road, Singapore 169608, Singapore; ^2^Department of Radiology, Northern Imaging Victoria, Epping, Melbourne, Australia; ^3^Department of Urology, Singapore General Hospital, Singapore.; ^4^Department of Pathology, Singapore General Hospital, Singapore

**Keywords:** MR Imaging, Urinary, Prostate, Neoplasms-Primary, Focal Therapy, Prostate Cancer, MRI, Surveillance, Tumor Recurrence

## Abstract

In this era of personalized precision medicine, the accuracy of multiparametric MRI (mpMRI) and targeted biopsy in helping detect low-volume clinically significant prostate cancer has rekindled interest in focal therapy for primary prostate cancer. Such therapy may reduce the debilitating morbidity of radical whole-gland treatment. Post–focal therapy mpMRI surveillance is critical for assessing oncologic efficacy. Radiologists interpreting post–focal therapy mpMRI must be familiar with expected posttreatment changes and pitfalls in assessing posttreatment recurrence. In this review, the authors present their experience with mpMRI before and after focal therapy. While cryotherapy and irreversible electroporation are the primary modalities of focal therapy offered in their institution, the authors aim to provide a comprehensive overview of the more common focal therapy modalities in use. Pertinent considerations of mpMRI in pretreatment patient selection and treatment planning are discussed. The recently proposed standardized post–focal therapy assessment systems, Prostate Imaging after Focal Ablation (ie, PI-FAB) and Transatlantic Recommendations for Prostate Gland Evaluation with MRI after Focal Therapy (ie, TARGET), as well as pearls and pitfalls in the detection of tumor recurrence and medium- and long-term mpMRI surveillance of the post–focal therapy prostate, are also discussed. This review aims to provide a valuable reference for radiologists involved in the care of patients in the evolving field of prostate cancer focal therapy.

**Keywords:** MR Imaging, Urinary, Prostate, Neoplasms-Primary, Focal Therapy, Prostate Cancer, MRI, Surveillance, Tumor Recurrence

Published under a CC BY 4.0 license.

**Summary**

Radiologists play a crucial role in pretreatment selection and posttreatment surveillance of patients undergoing focal therapy for primary prostate cancer.

**Essentials**

■ In patients with prostate cancer being considered for focal therapy, pretreatment considerations include accurate risk stratification through tumor detection and staging and precise determination of tumor location and volume.
■ Focal therapy distorts the prostate and alters the signal intensity of treated and untreated parenchyma; therefore, dynamic contrast-enhanced MRI sequence is the dominant sequence in assessing tumor recurrence.
■ Following focal therapy, multiparametric MRI is a crucial adjunct to serum prostate-specific antigen level monitoring and histologic sampling in medium- and long-term surveillance.

## Oncologic Basis of Focal Therapy

Whole-gland treatment approaches such as radical prostatectomy and radiation therapy have long been regarded as the reference standard of definitive treatment of prostate cancer, with robust long-term oncologic outcome data (1). However, these interventions are associated with substantial morbidities, including urinary incontinence and erectile dysfunction (2).

To reduce overtreatment of prostate cancer, active surveillance was introduced 2 decades ago, where patients with low-risk or favorable intermediate-risk disease are closely monitored and undergo definitive treatment only when more aggressive disease manifests. While active surveillance is a reasonable option for low-grade, localized cancers, controversy remains over whether patients with intermediate-risk disease are suitable candidates. Besides the risk of progressive disease, active surveillance is disadvantageous, as patients are subjected to frequent follow-up visits, repeat imaging and biopsies, and anxiety and financial strain (2).

Consequently, various focal therapy options have been explored in an attempt to find a middle ground between whole-gland therapy and active surveillance. Considerable evidence suggests that although prostate cancer is often multifocal, it is the pathologic characteristics of the largest or most aggressive cancer focus (index lesion) that determines tumor progression and metastasis risk (3,4). It may therefore be sufficient to treat the index lesion and adopt active surveillance for the remaining low-risk lesions. The challenge of focal therapy is to improve quality of life without jeopardizing oncologic control and to focus treatment on the tumor while minimizing injury to the rest of the prostate, particularly the neurovascular bundles, bladder neck, and urethral sphincter.

Due to the paucity of reliable evidence on long-term efficacy and lack of randomized trials supporting these emerging treatment strategies, current European Association of Urology and American Urological Association guidelines recommend focal therapy to be performed only in experienced centers within the context of a clinical trial or a well-designed prospective cohort study (5,6). However, short- and intermediate-term clinical data have shown promising oncologic and functional outcomes, supporting these strategies as potential standard-of-care options (7). These techniques have garnered much interest and have been fast gaining popularity among physicians and patients.

## Role of Multiparametric MRI in Prostate Cancer Surveillance After Focal Therapy

Serum prostate-specific antigen (PSA) testing is widely used for surveillance after whole-gland treatment. However, it is less reliable following focal therapy because of the substantial amount of residual prostatic parenchyma that will continue to produce PSA. False-positive PSA level bounces unrelated to tumor recurrence or residual disease can also occur (8).

Multiple guidelines advocate for routine protocol ablation zone biopsies after treatment regardless of MRI findings (9–11). However, these biopsies carry risks and have had poor patient compliance in previous focal therapy series (11,12).

The multifocality of prostate cancer explained by the concept of epigenetic field defect (13), together with the presence of substantial residual untreated gland, warrants a noninvasive and reliable surveillance modality. In the 2020 international multidisciplinary consensus for surveillance after focal therapy for localized prostate cancer, experts agreed that multiparametric MRI (mpMRI) is the preferred imaging modality to evaluate treatment response, as it is the most established. They acknowledged that this is an evolving field, however, and other imaging modalities may prove beneficial (11).

## Aim of Review

In this review, we present our experience with mpMRI before and after focal therapy. While cryotherapy and irreversible electroporation (IRE) are the primary modalities of focal therapy offered in our institution, we aim to provide a comprehensive overview and discussion of the more common focal therapy modalities in use. Early posttreatment appearance of the prostate varies based on focal ablative modality, but later posttreatment changes are similar across various modalities. Similar principles in long-term evaluation of tumor recurrence may therefore be applied.

We discuss pertinent considerations of mpMRI in pretreatment patient selection and treatment planning. While the Prostate Imaging and Reporting Data System (PI-RADS) has been transformative in standardizing mpMRI scoring for prostate cancer detection in treatment-naive patients, its use to assess the posttreatment prostate is inappropriate. Prostate Imaging after Focal Ablation (PI-FAB) and Transatlantic Recommendations for Prostate Gland Evaluation with MRI after Focal Therapy (TARGET) are recently proposed MRI scoring systems aimed to standardize mpMRI evaluation after focal therapy. We discuss potential applications of PI-FAB and TARGET, pearls and pitfalls in the detection of tumor recurrence, as well as medium- and long-term post–focal therapy mpMRI surveillance. A summary of this review may be found in the Table.

**Table:**
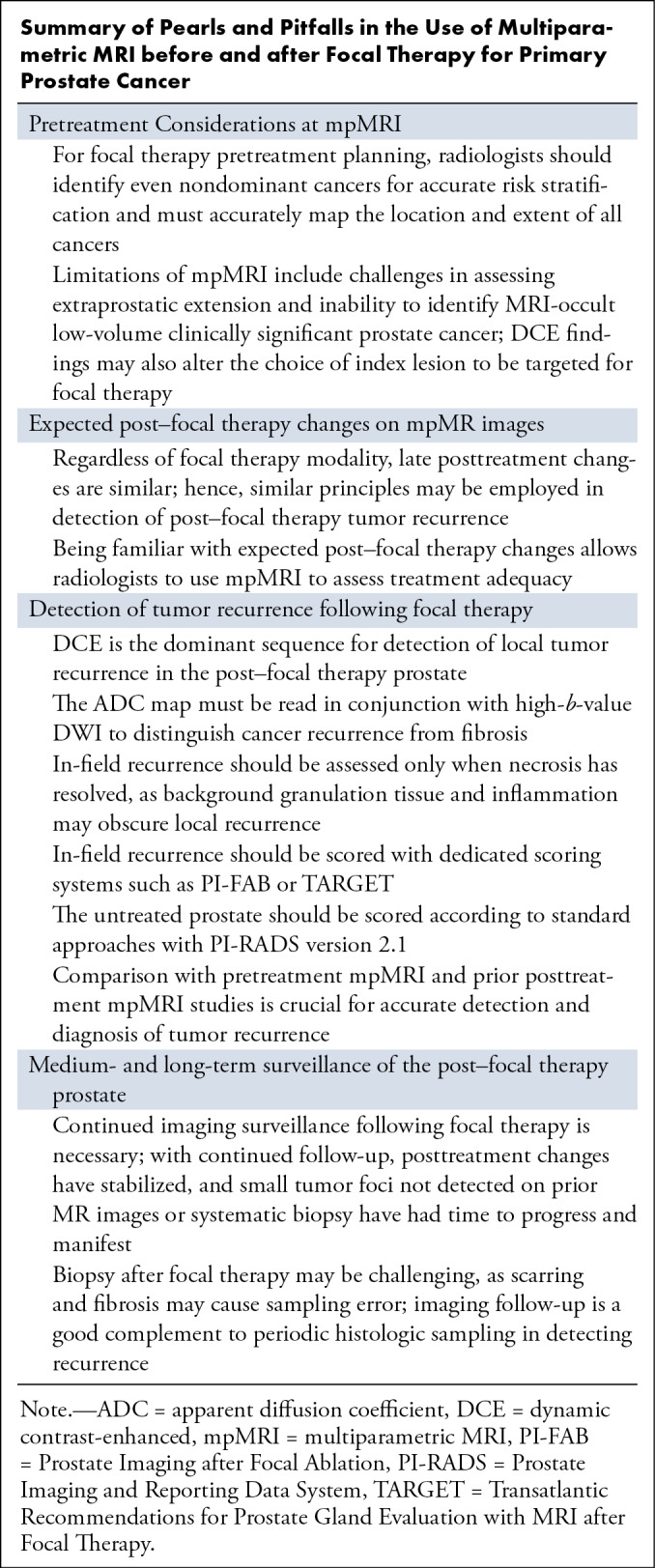
Summary of Pearls and Pitfalls in the Use of Multiparametric MRI before and after Focal Therapy for Primary Prostate Cancer

## Overview of Focal Therapy

### Focal Therapy: Methods

Focal therapy aims to treat only the part of the prostate that harbors clinically significant cancer, while preserving the rest of the gland. It relies on the use of various energies for local destruction of cancer cells, including high-intensity focused ultrasound (HIFU), cryotherapy, focal laser ablation, IRE, photodynamic therapy, and locally injected cytotoxic drugs.

In cryotherapy, cancers are treated by repetitive cycles of freezing and thawing, inducing cell rupture and death (14). Cryoprobes are placed into the prostate transperineally under transrectal US guidance, and the tumor is cooled to a minimum lethal temperature of −40 °C (15). Monitoring probes at the urethral sphincter and prostatic apex and adjacent to the neurovascular bundles provide thermal control. A urethral warming catheter reduces urethral injury and sloughing (15).

In HIFU, focal ultrasound is used to heat the target area, generating a cavitation effect and causing coagulation necrosis (16).

In IRE, electrodes are placed transperineally under US guidance. Electrical pulses are delivered between the electrode pairs, increasing the permeability of the cell membranes and resulting in tissue ablation (17).

In focal laser ablation, a directed laser beam is used to thermally destroy prostatic tissue under MRI guidance. MR thermometry allows real-time temperature monitoring and adjustment (18).

### Focal Therapy: Outcomes

The location of cancer within the prostate is key in predicting the type and frequency of complications. Patients with cancers located near the urethra, bladder neck, and apex are at higher risk of developing postoperative irritative and obstructive lower urinary tract symptoms. Cancers located near the neurovascular bundles with capsular contact require extended ablation times, which may negatively impact erectile function recovery (19). Prostate size is also a consideration: Particularly large prostates may not be suitable for some focal therapy modalities or treatment templates, and such patients are more prone to developing postoperative lower urinary tract symptoms (20). The amount of treated tissue also affects toxicity, with increased treated prostatic tissue associated with increased postoperative complications (21).

When compared with whole-gland treatment, focal therapy is associated with substantially fewer adverse events and improved preservation of genitourinary function (21). In a recent review and meta-analysis of focal therapy outcomes following cryotherapy, HIFU, and IRE, the majority of cohorts reported low and moderate impact on sexual function (45.7% and 48.6%, respectively), while a small cohort (5.7%) reported severe impact. For urinary function outcomes, an overwhelming majority (97.1%) reported low impact, and only one cohort (2.9%) reported moderate impact. No severe impact on urinary function was reported (22).

The most common postoperative complications are mild and usually occur within 30 days following focal therapy, including hematuria, infections, and catheter-related issues such as discomfort, pain, and urethral sloughing (23). In the Partial Ablation versus Radical Treatment (ie, PART) randomized control trial comparing radical prostatectomy with focal ablation by HIFU, at 6 months no patients who had undergone HIFU reported the need to use pads, as compared with approximately 60% of patients who had undergone radical prostatectomy (24). A combined analysis of three prospective development trials evaluating erectile dysfunction after focal HIFU demonstrated a complete return to baseline function at 1 year (25).

## Considerations in Focal Therapy Patient Selection and Pretreatment Planning

### Pretreatment Workup

Success in focal therapy is contingent on accurate pretreatment patient selection and precise disease localization. Focal therapy is considered ideal for localized, discrete, small-volume clinically significant cancers in intermediate-risk patients with good life expectancy, provided the cancers can be accurately targeted and the energy source is able to completely ablate the lesion with an appropriate margin. Tumor foci of less than 1.5 mL on mpMR images or less than 20% of total prostate volume are suitable for focal therapy; tumor foci up to 3 mL in size or 25% of total prostate volume may also be suitable if localized to one hemigland (26). Remaining small-volume Gleason grade 3 + 3 untreated areas are deemed acceptable and can be monitored with active surveillance (26).

Each patient considered for focal therapy is required to undergo a rigorous diagnostic workup. Following prostate mpMRI, targeted and systematic prostate biopsy or mapping biopsy is necessary to accurately delineate the margins of the index lesion and rule out with high reliability MRI-occult clinically significant lesions (27,28). While the optimal number of systematic biopsy cores is unclear in the era of mpMRI-targeted biopsy, a comprehensive systematic biopsy is recommended in focal therapy planning. A recent study by Lee et al (29) has shown that reducing the number of systematic biopsy cores may potentially reduce detection of clinically significant prostate cancer and limit the oncologic efficacy of focal therapy.

Considerations in patient selection for focal therapy are described below.

### Prostate Size

Older-generation HIFU devices were limited to prostate glands with volume smaller than 40 mL because of limitations in focal distance length (30). Treatment may also be challenging when the focal point falls outside the prostate in smaller glands, especially for lesions in the peripheral zone (31). Pretreatment with androgen deprivation therapy or transurethral resection of the prostate may be considered to reduce large glands to effective size (32).

Cryotherapy must also be used with caution in tumors located in smaller prostates, as the ice ball formed during the procedure may extend into the adjacent neurovascular bundle or urethra, increasing the risk of injury and damage (14). Other modalities like IRE and focal laser ablation do not appear to be restricted by prostate volume.

### Tumor Location

Tumor location is important in selecting an appropriate focal therapy modality. For posterior tumors, HIFU is ideal because of its transrectal approach, shorter focal distance, and precise contouring of the target area (31). Caution must be exercised when treating posterior lesions with cryotherapy because of the risk of inadvertently ablating the adjacent neurovascular bundles, which may negatively impact recovery of erectile function (19).

For anterior tumors, cryotherapy is ideal because of its transperineal approach that poses little risk of rectal injury, with negligible fistula rates (32).

For periurethral tumors, IRE may be considered to minimize postoperative irritative and obstructive lower urinary tract symptoms. Cryotherapy is not ideal, as the urethral warming catheter may prevent periurethral tissue from reaching the minimal lethal freezing temperature, resulting in undertreatment (32).

For apical tumors, focal brachytherapy has been shown to demonstrate extremely low urethral toxicity (33). Other energy modalities have the potential to cause damage to the urethral sphincter, which may result in incontinence without achieving oncologic control (32).

### Anatomic Abnormalities

Ablative modalities employing a transrectal approach, such as HIFU and transrectal US-guided cryoablation, cannot be used in cases of rectal abnormalities such as congenital defects, prior anorectal resection, or postradiation strictures. Newer techniques employing a transperineal route without transrectal US control but with MR guidance, such as focal laser ablation, cryotherapy, and brachytherapy, may be helpful in these cases (31).

### Pitfall: Challenges in Accurate Risk Stratification for Pretreatment Planning

Unlike whole-gland treatment, only the index lesion is treated in focal therapy, while small-volume low-grade lesions may be monitored with active surveillance. Therefore, radiologists should identify all possible cancers to accurately triage the patient for risk stratification and appropriate counseling. Radiologists also play a crucial role in accurately mapping the location and extent of all cancers, as this impacts treatment planning and determines the most appropriate mode of focal therapy.

However, limitations of mpMRI in pre–focal therapy assessment include inability to identify MRI-occult low-volume clinically significant prostate cancer, underestimation of cancer burden, and challenges in assessing extraprostatic extension.

When correlated with whole-mount pathology prostatectomy specimens, mpMRI has been reported to miss at least one clinically significant prostate cancer in a third of patients overall and in close to half of patients with multifocal lesions. The vast majority of mpMRI-missed lesions were small lesions less than 1 cm (34). Moderate per-lesion sensitivity of mpMRI is an important limitation in accurate risk stratification of patients for focal therapy.

mpMRI consistently underestimates the size and extent of prostate cancer, particularly for larger tumors and tumors containing high-grade cancer (35), as well as tumor borders, which are usually irregular and not as circumscribed as visualized at mpMRI. Priester et al (36) concluded that up to 80% of cancer volume may lie outside of the visible region of interest seen at mpMRI. The treatment margin acceptable for primary focal therapy is reported to be circumferentially 5–15 mm surrounding the lesion as it appears at imaging (36,37). Le Nobin et al (37) found that on analysis of software-assisted MRI and prostatectomy coregistration, a margin of at least 9 mm is required to achieve complete treatment of the entire histologic tumor during focal therapy.

**Figure 1:**
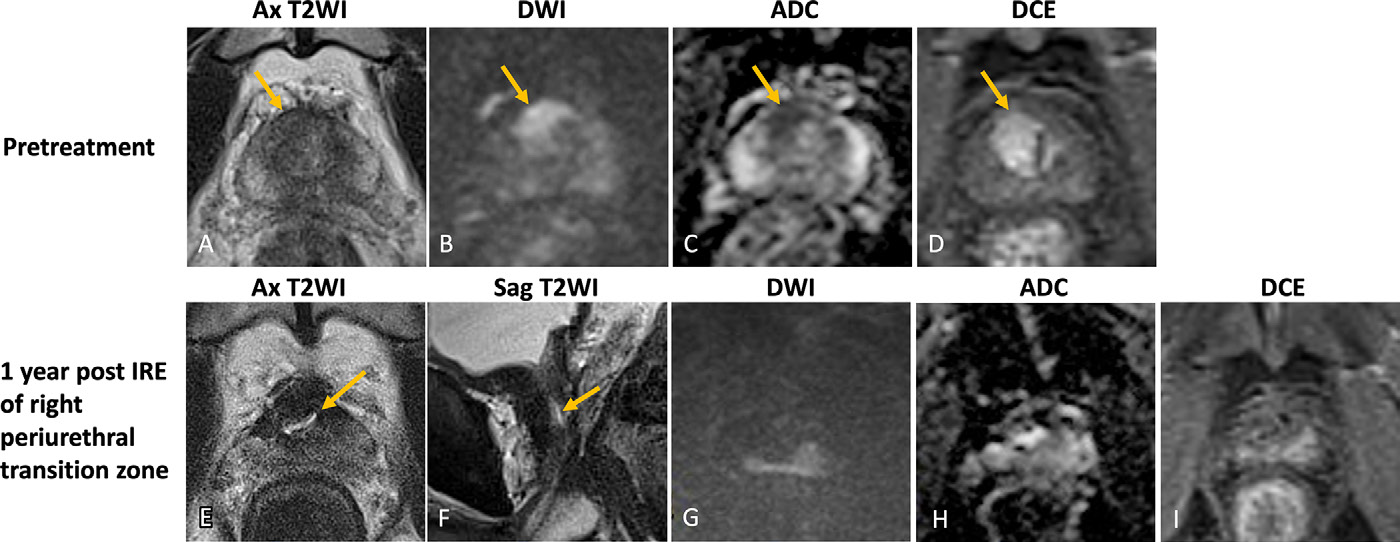
Images in a 67-year-old male patient with biopsy-proven Gleason grade 3 + 4 prostate cancer. Case illustrates pretreatment DCE upstaging tumor and altering clinical management. **(A–D)** Images from pretreatment MRI. **(A)** Axial T2-weighted image, **(B)** high-*b*-value DW image, and **(C)** ADC map demonstrate a T2-weighted hypointense lenticular subcapsular lesion in the right anterior transition zone midgland with restricted diffusion (arrow). Based on biparametric MRI findings, this lesion is smaller than 1.5 cm and would thus be graded PI-RADS 4. However, on **(D)** DCE images, the enhancing area is found to be larger than 1.5 cm and extending to the periurethral transition zone (arrow). Findings from targeted biopsy of the lenticular subcapsular lesion and systematic biopsy of the periurethral transition zone adjacent to the lesion were positive for Gleason grade 3 + 4 prostate cancer. This patient had originally been considered for focal cryoablation. However, given the periurethral location of the lesion, cryoablation would be ineffective, as the urethral warming catheter would prevent periurethral tissue from reaching the minimum lethal freezing temperature. Hence, IRE was employed instead. **(E–I)**. MR images 1 year after IRE of the right periurethral transition zone. **(E)** Axial and **(F)** sagittal T2-weighted images show hypointense scarring with a cystic cavity at the treatment site (arrow), representing a urinoma, characteristic of post-IRE changes. **(G)** High-*b*-value DW image and **(H)** ADC map demonstrate signal void at the treatment site and no residual restricted diffusion is seen. **(I)** DCE image shows no focus of enhancement to suggest residual or recurrent tumor. Post-IRE MRI finding is PI-FAB 1/TARGET 1. Findings from 1-year post–focal therapy surveillance targeted biopsy at the treatment site were negative, with no residual cancer. ADC = apparent diffusion coefficient, Ax = axial, DCE = dynamic contrast-enhanced, DWI = diffusion-weighted imaging, IRE = irreversible electroporation, PI-FAB = Prostate Imaging after Focal Ablation, PI-RADS = Prostate Imaging Reporting and Data System, T2WI = T2-weighted imaging, TARGET = Transatlantic Recommendations for Prostate Gland Evaluation with Magnetic Resonance Imaging After Focal Therapy.

While there is clear underestimation of cancer size on diffusion-weighted imaging (DWI) studies and T2-weighted images, existing literature suggests that enhancement on dynamic contrast-enhanced (DCE) images may more accurately reflect true tumor extent when correlated with histopathology (38). PI-RADS 4 lesions may be found to be larger on DCE images and be upgraded to PI-RADS 5. DCE findings may hence alter the choice of index lesion to be targeted for focal therapy, with important implications in treatment planning (39) (Fig 1). Ultimately, the true benefit of DCE imaging has been shown to be variable, and a key determinant of DCE accuracy is reader experience (40).

In this light, given the current rising interest in biparametric non–contrast-enhanced screening prostate MRI, for patients considering focal therapy, one may consider repeating pretreatment MRI with contrast media versus using targeted and perilesional biopsies to more accurately delineate the margins of the treatment zone.

## mpMRI Protocol for Scanning the Prostate after Focal Therapy

Following focal therapy, the prostate gland should be imaged using an mpMRI protocol that includes T2-weighted imaging, DWI, and DCE sequences. The 2024 TARGET international consensus advocates that given the importance of DCE in the posttreatment setting, a biparametric protocol that omits DCE sequences should not be used (41). Adequate imaging can be performed at either 1.5 T or 3.0 T, but 3.0 T is preferred. An endorectal coil is neither mandatory nor preferred at either field strength. Sequence technical parameters should at least match PI-RADS version 2.1 standards, but ultimately, high-quality images are of paramount importance, and parameters should be optimized for the scanner available (41).

There is substantial variability in the literature on the timing and frequency of surveillance mpMRI after focal therapy. The 2020 international consensus on surveillance following focal therapy recommends initial postoperative imaging within 6 months following focal therapy, with subsequent mpMRI 12 months later (11). The 2024 TARGET international consensus recommends first protocol surveillance mpMRI at 12 months to allow sufficient time for treatment-induced coagulative necrosis and inflammation, which may mask recurrent tumor, to subside and for PSA kinetics to stabilize (41). At our institution, the first surveillance mpMRI is performed 12 months after focal therapy.

## Expected Posttreatment Changes Following Focal Cryotherapy and Other Focal Therapy Modalities

### Early Postablation Changes (Less than 6 Months after Focal Therapy)

Immediate and early posttreatment changes are typically not visualized in day-to-day clinical practice, as follow-up surveillance mpMRI is usually performed beyond the initial 6 months following treatment to minimize posttreatment changes that may mimic or obscure tumor recurrence.

Immediate and early posttreatment appearance of the prostate varies based on the focal ablative modality used. For example, in the early post–focal cryotherapy period, the ablation zone appears hyperintense with hypointense rim on T1-weighted images, T2-weighted images, and DW images. The hyperintense signal is due to central necrosis, hemorrhage, and inflammatory response, while the hypointense rim reflects surrounding granulation tissue or hemosiderin deposition (42). Peripheral enhancement is common and may be due to granulation tissue at the ablation margins or transient inflammatory response in the surrounding prostatic parenchyma (42). Occasionally, internal enhancement may be observed within the ablation zone. This is due to transient inflammation rather than residual tumor and should resolve by 6 months (42). The posttreatment ablation site may be indistinguishable from an abscess (which can demonstrate variable T1-weighted imaging and T2-weighted imaging appearance), and context is crucial to come to the correct diagnosis in this setting.

With HIFU, a "double-ring sign" is typically observed 1–3 months following treatment. This appearance is unique to HIFU and constitutes thin curvilinear enhancement along either side of the T2-weighted imaging hypointense prostate gland capsule (43).

With IRE, prostate volume increases substantially immediately following treatment probably due to swelling from posttreatment inflammation (44). At 1 month following treatment, the treated area demonstrates heterogeneous signal intensity on T2-weighted images, focal hyperintense signal on T1-weighted images because of hemorrhage, and areas of nonenhancement (44).

With focal laser ablation, the treated area is better delineated than with other modalities because of the targeted nature of the procedure (45). Following treatment, the ablated region demonstrates heterogeneous T2-weighted hypointense signal with restricted diffusion (45).

### Late Postablation Changes (at Least 6 Months after Focal Therapy)

All these minimally invasive procedures for treating localized prostate cancer are highly effective and eventually lead to fibrosis of the treated area. By 6–12 months after treatment, regardless of focal therapy modality employed, the posttreatment prostate will demonstrate scarring and fibrosis with atrophy, decreased T2-weighted imaging signal intensity, and hypointense signal at DWI and apparent diffusion coefficient (ADC) mapping (46). While early posttreatment appearance of the prostate varies across focal ablative modalities, late posttreatment changes are similar. Hence, similar principles may be applied in long-term evaluation of tumor recurrence regardless of focal therapy modality.

**Figure 2:**
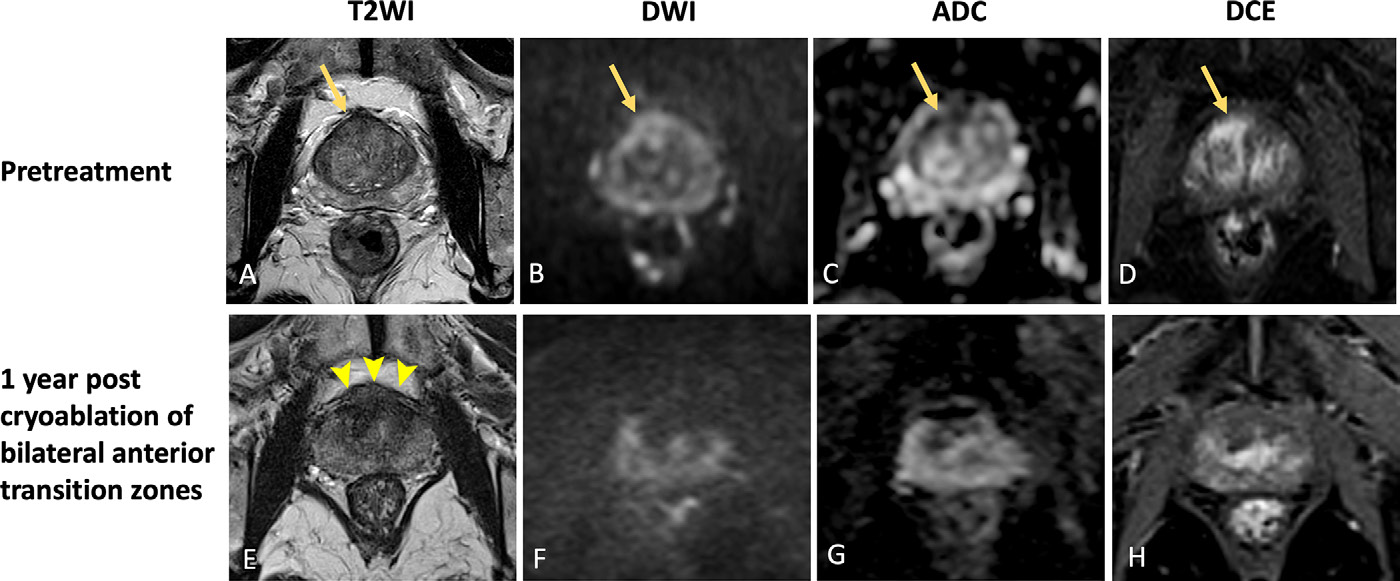
Images in a 77-year-old male patient with prostate cancer. Case illustrates adequate treatment with expected post–focal therapy changes. **(A–D)** Images from pretreatment MRI. **(A)** Axial T2-weighted image, **(B)** high-*b*-value DW image with **(C)** corresponding ADC map, and **(D)** DCE image show focal T2-weighted hypointense lesion with restricted diffusion and early enhancement in the anterior transition zone to the right of the midline (arrow). The lesion was assigned PI-RADS category 5. Targeted biopsies showed Gleason gradeç 3 + 4 prostate cancer. Patient underwent focal cryoablation of bilateral anterior transition zones. **(E–H)** MR images 1 year after focal cryoablation. **(E)** Axial T2W image shows hypointense scarring with linear margins in bilateral anterior transition zones (arrowheads) with associated volume loss, capsular retraction, and adjacent periprostatic fibrosis. Previously seen PI-RADS 5 lesion is no longer visualized. **(F)** High-*b*-value DW image and **(G)** ADC map demonstrate signal void at the treatment site with no residual restricted diffusion. **(H)** DCE image shows no focus of enhancement to suggest residual or recurrent tumor. ADC = apparent diffusion coefficient, DCE = dynamic contrast-enhanced, DWI = diffusion-weighted imaging, PI-RADS = Prostate Imaging Reporting and Data System, T2WI = T2-weighted imaging.

Being familiar with expected posttreatment changes also allows radiologists to use post–focal therapy mpMRI to assess treatment adequacy. With adequate treatment (Fig 2), mpMRI should demonstrate linear T2-weighted hypointense signal and volume loss at the treated site in keeping with fibrosis. DWI typically demonstrates signal void at the treated site, and no residual focus of diffusion restriction or enhancement should be observed.

**Figure 3:**
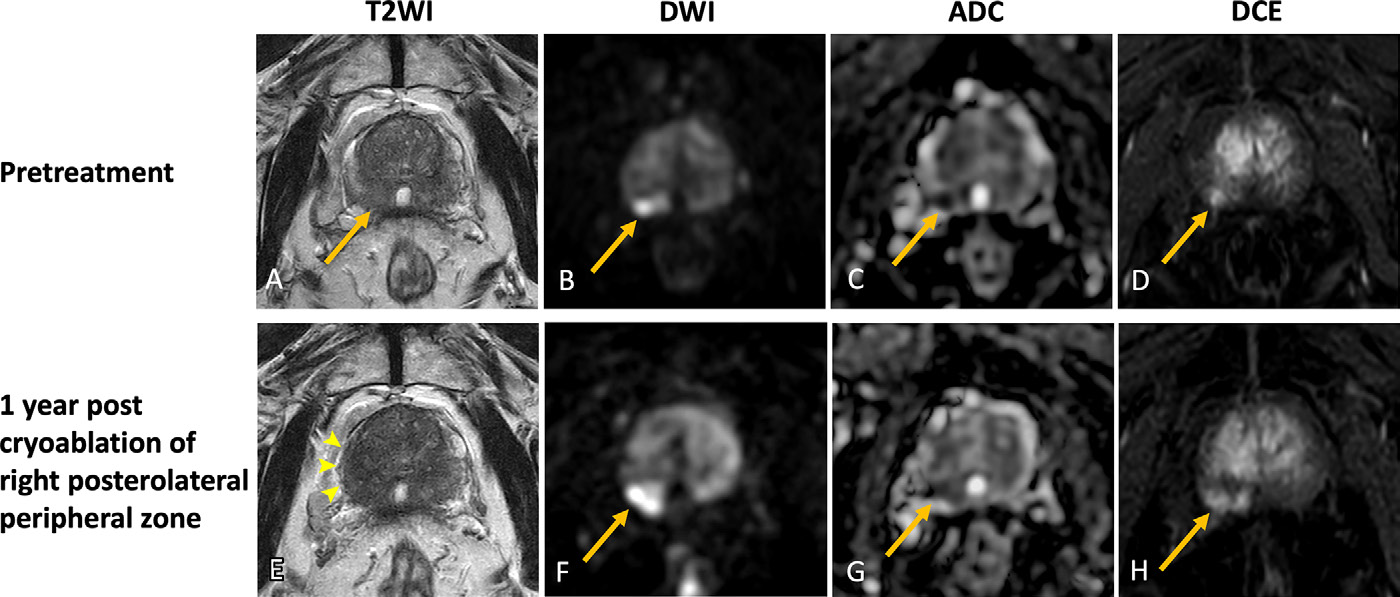
Images in a 76-year-old male patient with prostate cancer. Case illustrates treatment failure with in-field residual tumor. **(A–D)** Images from pretreatment MRI. **(A)** Axial T2-weighted image, **(B)** high-*b*-value DW image with **(C)** corresponding ADC map, and **(D)** DCE image show focal hypointense lesion with restricted diffusion and enhancement (arrow) in the right posterior peripheral zone. Lesion was assigned PI-RADS category 4. Targeted biopsies showed Gleason grade 3 + 4 prostate cancer. Patient underwent focal cryoablation of the right posterolateral peripheral zone. **(E–H)** MR images 1 year after focal cryoablation. **(E)** Axial T2-weighted image shows ill-defined intermediate to low signal intensity at the site of the tumor (arrowheads) with absence of volume reduction, raising concern for inadequate treatment. Furthermore, **(F)** axial high-*b*-value DW image, **(G)** ADC map, and **(H)** DCE image show that the lesion in the right posterior peripheral zone at the ablation zone has increased in size (arrow) with persistent restricted diffusion and enhancement. Post–focal therapy MRI finding is PI-FAB 3/TARGET 5. Histopathology revealed Gleason grade 3 + 4 in-field residual tumor. In this case, the initial tumor could have been preferentially treated with another modality such as high-intensity focused ultrasound, given its small size and posterior location near the neurovascular bundle. ADC = apparent diffusion coefficient, DCE = dynamic contrast-enhanced, DWI = diffusion-weighted imaging, PI-FAB = Prostate Imaging after Focal Ablation, PI-RADS = Prostate Imaging Reporting and Data System, T2WI = T2-weighted imaging, TARGET = Transatlantic Recommendations for Prostate Gland Evaluation with Magnetic Resonance Imaging After Focal Therapy.

With inadequate treatment, however, the treated site may demonstrate indistinct T2-weighted intermediate hypointense signal and lack of volume loss. The treatment site may also demonstrate persistent focal diffusion restriction and/or enhancement, indicating residual tumor (Fig 3).

## Tumor Recurrence Following Focal Therapy

### Location of Recurrent Tumor

Tumor recurrence following focal therapy can be categorized into in-field and out-of-field recurrence. In-field recurrence, which refers to recurrence within the ablation zone or at the margins of ablation, signifies inadequate treatment. Out-of-field recurrence refers to cancer detected away from the treatment site. These lesions may imply “selection failure,” especially if clinically significant prostate cancer is identified within 12–18 months of focal therapy (11), suggesting that these lesions were missed at initial evaluation. Many of these lesions may have been small volume or occult at initial mpMRI and therefore not sampled with conventional systematic biopsy. These initially MRI-occult de novo cancers may have progressed and become apparent during post–focal therapy surveillance, once again demonstrating the critical role of mpMRI in imaging surveillance (11).

### Appearance of Recurrent Tumor

International consensus incorporates DCE as a major sequence and DWI and T2-weighted imaging as joint minor sequences in assessing recurrent tumor in the post–focal therapy setting (41,47). Focal nodular strong early contrast enhancement is most suspicious for tumor recurrence (11,41) and reflects a tumor-like morphology with malignant vascular perfusion and permeability as seen in neoangiogenesis (48). In contrast, areas of focal nodular mild early enhancement that appear less intense than other areas of the prostate, or areas of linear early enhancement, are equivocal (41), and follow-up may be helpful to determine if such findings are due to treatment-induced inflammation.

From our experience, and also as recommended in international consensus, DWI plays a more important role than T2-weighted imaging in post–focal therapy MRI assessment. A combination of these features is suggestive of recurrence: T2-weighted imaging hypointense signal and a combination of hypointense signal on ADC maps, with hyperintense signal on high-*b-*value (*b* value of ≥ 1400 sec/mm^2^) DW images in the treated region (11).

## Scoring the Prostate Following Focal Therapy

### Pearl: For In-Field Recurrence, DCE Is Considered the Dominant Sequence for Detection of Local Tumor Recurrence

Both primary and recurrent prostate cancer demonstrate similar imaging characteristics: T2-weighted imaging hypointense signal, high signal intensity on high-*b*-value DW images coupled with low signal intensity on ADC maps, and early enhancement on DCE images. Following focal therapy however, T2-weighted imaging and DWI, the dominant sequences according to PI-RADS guidelines (49), are compromised by the presence of posttreatment fibrosis, which demonstrates hypointense signal on T2-weighted images and ADC maps. DCE hence assumes a dominant role for the detection of locally recurrent tumor in both the peripheral and transition zones in the posttreatment setting (47). With PI-RADS version 2.1, DCE is a minor sequence used only to upgrade equivocal peripheral zone lesions (49). Thus, the use of PI-RADS version 2.1 is inappropriate for the scoring of the treated gland.

To address this, two scoring systems to assess the likelihood of local tumor recurrence at post–focal therapy prostate mpMRI on a per-lesion basis have recently been proposed: PI-FAB, a three-point scoring system proposed by Giganti et al (47), and TARGET, a five-point scoring system proposed by Light et al (41).

### PI-FAB Scoring System

For a lesion demonstrating hypointense signal on T2-weighted images and high-*b*-value DWI with no enhancement at the site of the original tumor, this likely represents fibrosis and is assigned PI-FAB score 1. mpMRI findings should be considered in conjunction with the patient’s clinical picture. For physically fit patients undergoing active treatment, continued monitoring is recommended.

For a lesion demonstrating focal enhancement with hypointense signal on T2-weighted images and high-*b*-value DW images:

1. If it is linear and not at the site of the original tumor, this likely represents a vessel or inflammation and is assigned PI-FAB score 1. Continued monitoring is recommended.
2. If it is an enhancing focus measuring 3 mm or less, and at the site of the original tumor, it is assigned PI-FAB score 2. Assessment of PSA kinetics is recommended, and biopsy should be considered if PSA level is rising. Otherwise, follow-up mpMRI should be performed in 1 year’s time.
3. If the enhancing focus is greater than 3 mm in size and is within or at the margin of the treatment site, or if it is a previously known PI-FAB score 2 focus that is now larger, it is assigned PI-FAB score 3 and biopsy is recommended.

For a lesion demonstrating focal enhancement and hyperintense signal at high-*b*-value DWI and hypointense signal at T2-weighted imaging, this is highly suspicious for residual or recurrent cancer and is assigned PI-FAB score 3. Biopsy is recommended.

### TARGET Scoring System

The TARGET scoring system is a two-tier algorithm that employs DCE as a major sequence and DWI and T2-weighted imaging as joint minor sequences. Each sequence is individually assessed on a scale of 1 to 3, where 1 = nonsuspicious, 2 = equivocal, and 3 = suspicious. Using these scores, an overall score out of 5 is then calculated, where 1 = very low suspicion, 2 = low suspicion, 3 = equivocal, 4 = high suspicion, and 5 = very high suspicion. Recommendations are also provided for scoring of the DCE sequence, where focal nodular strong early enhancement is suspicious and is assigned score 3, focal nodular mild early enhancement or thin linear early enhancement or curvilinear early enhancement is equivocal and is assigned score 2, and no early enhancement or focal late enhancement or any other DCE finding not meeting the criteria for scores 2 or 3 is nonsuspicious and is assigned score 1.

### Discussion of PI-FAB and TARGET Scoring Systems

With both PI-FAB and TARGET, in-field recurrence should be assessed only when necrosis has resolved, as background granulation tissue and inflammation may otherwise obscure local recurrence. Furthermore, the untreated prostate should be scored according to standard approaches with PI-RADS version 2.1.

PI-FAB is a proposal based on experience with HIFU from a single center without a consensus process, while TARGET is based on systematic review and a multicenter international expert consensus meeting. PI-FAB is a three-point scale, whereas with TARGET, each sequence is assessed on a scale of 1 to 3 to calculate an overall score out of 5. Both scoring systems place DCE as the dominant base sequence for detection of tumor recurrence, followed by sequential evaluation of DWI and T2-weighted imaging. TARGET provides specific recommendations for the scoring of DCE sequences, while PI-FAB takes into consideration the size and growth of the enhancing focus on DCE images. PI-FAB provides next-step clinical recommendations based on the scores, while TARGET does not.

Both PI-FAB and TARGET have their drawbacks. Both rely heavily on DCE MRI, which may not be readily available in all clinical settings, such as in patients with renal impairment, and application may be limited in institutions where DCE is infrequently used or unavailable.

Furthermore, both PI-FAB and TARGET scoring systems place reduced emphasis on high-*b*-value DWI. Tumor and treated tissue both demonstrate hypointense signal on ADC map and T2-weighted images, so high-*b*-value DWI may be useful to distinguish the two. DWI may also occasionally serve as the leading sequence above DCE, especially in the transition zone when differentiating tumor from other enhancing nodules in benign prostatic hyperplasia (50). Moreover, a focus with marked focal restricted diffusion, but without early strong enhancement, is scored low suspicion with both scoring systems. There are currently insufficient data to determine if this is appropriate; further studies are needed to validate both scoring systems.

Another point of discussion that has been raised with PI-FAB is the potential challenge of applying a three-point score in a community accustomed to a five-point scoring system, as with PI-RADS for scoring the treatment-naive prostate (49), Prostate Imaging for Recurrence Reporting system (ie, PI-RR) following radical prostatectomy or radiation therapy (51), and Prostate Cancer Radiological Estimate of Change in Sequential Evaluation (PRECISE) for radiologic changes during active surveillance (52). Moreover, following focal therapy, PI-RADS version 2.1 is still applied to lesions outside the ablation zone. There may hence be difficulty combining a five-point system for the nonablated regions and a three-point system for the ablated regions (50). On the other hand, as pointed out by the authors themselves, studies have often merged the lowest and the highest scores, as these extreme values usually convey the same message (eg, radiologic progression for PRECISE 4 and 5). Combined with the clinical next-step recommendations, the three-point scoring system may therefore be more intuitive. Perhaps a system using only descriptors and no numbers may help to reduce confusion, such as in the Liver Imaging Reporting and Data System treatment response algorithm (ie, LI-RADS TRA), where treated lesions are categorized as nonevaluable, nonviable, equivocal, or viable (53).

While long-term validation studies assessing these scoring systems are needed, an initial retrospective study was performed by Gelikman et al (54). In a mixed cohort of 38 patients, the PI-FAB score demonstrated 93% sensitivity in detecting clinically significant recurrent prostate cancer, with specificity ranging from 54% to 63% and positive predictive value from 54% to 59% among expert radiologists. Moderate agreement (κ = 0.56) was observed.

Another study by Pausch et al (55) of 73 patients after undergoing HIFU showed lower sensitivity (43% and 14%) and positive predictive value (33% and 14%) among readers with different experience levels, but high specificity (87%–98%) and negative predictive value (88%–100%), with strong interreader agreement (Gwet AC1, 0.80–0.95).

In a study by Ferriero et al (56) of 43 patients after undergoing cryoablation, 12-month posttherapy mpMR images scored with PI-RADS were retrospectively reviewed by a single expert reader, and a PI-FAB score was assigned. Sensitivity, specificity, positive and negative predictive values, and accuracy were 83.3%, 91.3%, 71.4%, 95.4%, and 89.6% and 75%, 80.6%, 60%, 89.3%, and 79.1% for PI-RADS and PIFAB score, respectively. Concordance rate of the two scores was 85.7%, with a κ index of 0.68.

These findings highlight PI-FAB’s potential in post–focal therapy MRI. A possible explanation for the disparate values among the studies by Gelikman et al and Pausch et al in terms of specificity and sensitivity may be differences in reader experience impacting diagnostic accuracy (16 and 9 years of experience, both with 1000 prostate MRI procedures per year, in Gelikman et al vs 4 years and 1 year of experience, with 300 and 250 prostate MRI procedures per year, respectively, in Pausch et al). Posttreatment mpMRI is challenging to interpret, and the novelty of the PI-FAB scoring system implies a possible learning curve; consequently, the disparity in mpMRI interpretation will be even more evident in readers with less experience with prostate mpMRI. This further emphasizes the need for continued research on a larger scale and more practice cases as the use of focal therapy becomes more widespread and more radiologists gain familiarity and expertise in this evolving field.

Additionally, PI-FAB demonstrates high negative predictive values across all studies, ranging from 89.3% to 100%, regardless of reader experience. This suggests that a low PI-FAB score may effectively rule out clinically significant in-field recurrence, potentially reducing the need for routine protocol after focal therapy biopsies.

Of note, a recent study by Esengur et al (57) compared the use of TARGET and PI-FAB for detecting tumor recurrence at mpMRI after primary focal therapy. In a mixed cohort of 38 patients, 14 of whom had recurrent clinically significant prostate cancer, PI-FAB showed higher sensitivity (92.9% for both readers compared with 78.6% and 92.9% for TARGET), while TARGET showed higher specificity (79.2% and 62.5% compared with 62.5% and 54.2% for PI-FAB). Both systems demonstrated moderate interreader agreement (κ = 0.56 for PI-FAB and 0.57 for TARGET). These findings suggest that both scoring systems exhibit similar performance in diagnosing recurrent cancer following focal therapy and are practical in the clinical setting.

## Case Review Illustrating the Appearance and Scoring of Histologically Proven Recurrent and Residual Cancer

**Figure 4:**
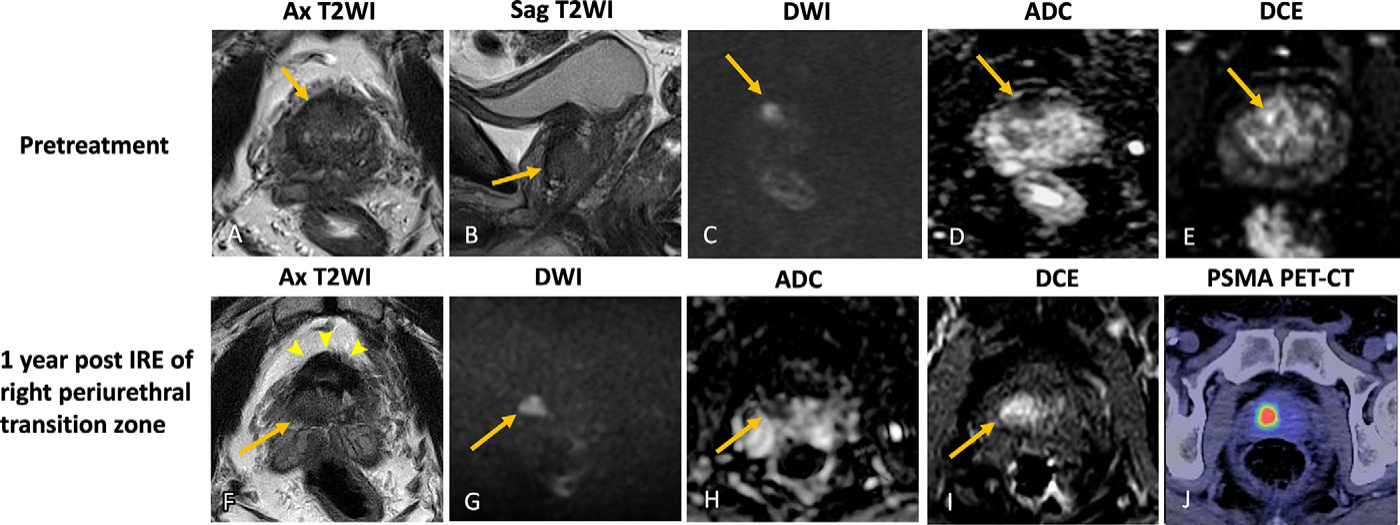
Images in a 75-year-old male patient with prostate cancer. Case illustrates in-field recurrence. **(A–E)** Images from pretreatment MRI. **(A)** Axial and **(B)** sagittal T2-weighted images, **(C)** high-*b*-value DW image, **(D)** ADC map, and **(E)** DCE image show a hypointense right transition zone periurethral tumor with restricted diffusion and enhancement (arrow). The lesion was assigned PI-RADS category 4. The patient underwent IRE of the right periurethral transition zone. **(F–J)** MR image 1 year after IRE. **(F)** Axial T2-weighted image shows hypointense scarring in the right anterior transition zone (arrowheads). However, a new lesion is seen at the margin of the treatment site (arrow) demonstrating **(F)** T2-weighted hypointense signal, **(G)** hyperintense signal on DW image, **(H)** hypointense signal on ADC map, and **(I)** focal enhancement on DCE image, suspicious for in-field recurrence. Post-IRE MRI finding is PI-FAB 3/TARGET 5. On **(J)** PSMA PET/CT image, the lesion corresponds with a PSMA-avid focus. Biopsy after IRE revealed Gleason grade 4 + 4 with tertiary Gleason pattern 5. The patient will be undergoing radical treatment. ADC = apparent diffusion coefficient, Ax = axial, DCE = dynamic contrast-enhanced, DWI = diffusion-weighted imaging, IRE = irreversible electroporation, PI-FAB = Prostate Imaging after Focal Ablation, PI-RADS = Prostate Imaging Reporting and Data System, PSMA = prostate-specific membrane antigen, Sag = sagittal, T2WI = T2-weighted imaging, TARGET = Transatlantic Recommendations for Prostate Gland Evaluation with Magnetic Resonance Imaging After Focal Therapy.

**Figure 5:**
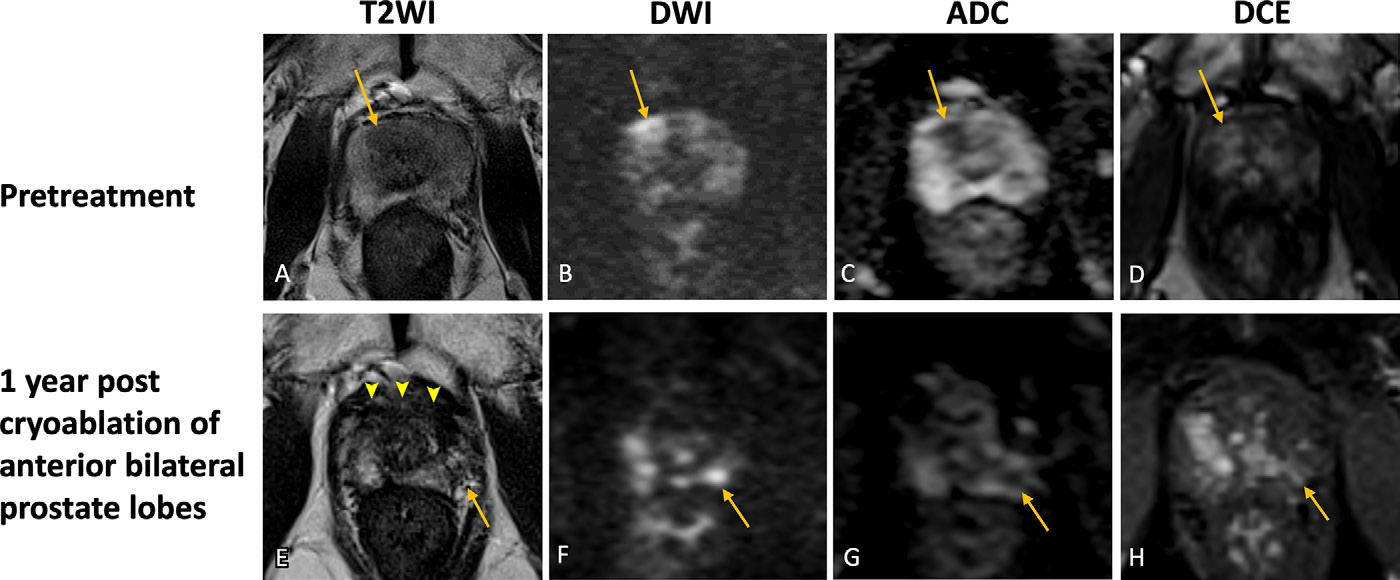
Images in a 77-year-old male patient with prostate cancer. Case illustrates out-of-field tumor recurrence. **(A–D)** Images from pretreatment MRI. **(A)** Axial T2-weighted image, **(B)** high-*b*-value DW image, **(C)** ADC map, and **(D)** DCE image show a hypointense lesion in the right anterior peripheral zone midgland with restricted diffusion and enhancement (arrow). The lesion was assigned PI-RADS category 4. The patient underwent cryoablation of the anterior aspect of bilateral prostate lobes. **(E–H)** MR images 1 year after focal cryoablation. **(E)** Axial T2-weighted image demonstrates volume loss, capsular retraction, and hypointense scarring in bilateral anterior peripheral zones (arrowheads) in keeping with expected postablation changes. No focus of enhancement or restricted diffusion is seen in the postablation zone to suggest residual tumor. In the left posterior peripheral zone apex outside of the ablation zone, there is a new lesion (arrow) with **(E)** T2-weighted hypointense signal, **(F)** hyperintense signal on high-*b*-value DW image, and **(G)** hypointense signal on ADC map with **(H)** enhancement, suspicious for tumor recurrence. Post–focal therapy MRI findings are PI-RADS score 4. Histopathology revealed Gleason grade 3 + 4 out-of-field recurrence. ADC = apparent diffusion coefficient, DCE = dynamic contrast-enhanced, DWI = diffusion-weighted imaging, PI-RADS = Prostate Imaging Reporting and Data System, T2WI = T2-weighted imaging.

In the following cases, we showcase our experience of post–focal therapy histopathologically proven clinically significant recurrent prostate cancer determined via targeted and systematic MRI/US fusion biopsy in a cohort of patients from a prospective phase II trial (CNIG17nov018 and TA20nov-0011) (58). The cancers are scored using the proposed PI-FAB and TARGET systems for in-field recurrence (Fig 4) and PI-RADS version 2.1 for out-of-field recurrence (Fig 5).

### Pearl: Subtraction Sequences May Be Helpful for Accurate Assessment of the Ablation Zone, as the Treated Tissue May Demonstrate Inherent T1-weighted Imaging Hyperintense Signal

After focal cryoablation, the ablation zone typically demonstrates T1-weighted hyperintense signal due to coagulative necrosis, hemorrhage, and inflammation. As residual or recurrent tumor is most accurately detected on DCE images, subtraction sequences may play a role in avoiding confounding areas of spurious contrast enhancement and allow accurate assessment of the ablation zone (59).

### Pearl: Comparison with Pretreatment mpMRI and prior Posttreatment mpMRI Studies Are Crucial to Distinguish Posttreatment Changes from Tumor Recurrence

Comparison with most recent pretreatment and subsequent posttreatment studies is crucial to track changes over time and to detect residual or recurrent tumor. Posttreatment changes will resolve or stabilize with time; for example, indeterminate linear enhancement at the ablation zone due to treatment effect is expected to resolve at further imaging surveillance. On the other hand, residual tumor will persist and progress, and recurrent tumor will show interval development. Information including date, modality and location of ablation, pretreatment tumor burden and grade, as well as PSA kinetics, are relevant and should be made available whenever possible.

### Pitfall: Posttreatment Changes May Mimic Tumor Recurrence

Following focal therapy, granulation tissue or hemorrhage may alter MRI signal intensity characteristics. Granulation tissue may demonstrate a nodular appearance and T2-weighted hyperintense signal. Like recurrent tumor, it may demonstrate enhancement on early DCE images due to hypervascularity. However, unlike tumor recurrence, granulation tissue should not exhibit diffusion restriction.

Hemorrhage exhibits a variable appearance. Typically, it demonstrates mild restricted diffusion with progressive enhancement rather than early enhancement with washout, but there may be overlap. In cases of ambiguity, prostate-specific membrane antigen PET/CT imaging may be helpful to differentiate hemorrhage from cancer.

Treatment-induced inflammatory changes are a major mimic of tumor recurrence, especially at and adjacent to the ablation site in the first few months following focal therapy. Both inflammatory changes and recurrent tumor demonstrate T2-weighted hypointense signal with restricted diffusion and enhancement. Morphology may be helpful for differentiation, as tumor recurrence demonstrates focal nodular strong early enhancement, while treatment-induced inflammation demonstrates a more diffuse pattern of enhancement (41).

For these reasons, international consensus recommends performing first protocol mpMRI at least 6 months after focal therapy to minimize posttreatment changes that may otherwise mask or mimic recurrent cancer (11,41). Continued follow-up imaging is also useful to allow posttreatment inflammation and fibrosis to stabilize and tumor recurrence to progress and manifest.

### Pearl: In the Post–Focal Therapy Setting, the ADC Map Must Be Read in Conjunction with High-*b*-Value DWI to Distinguish Cancer Recurrence from Fibrosis

Studies in treatment-naive prostates have shown that tumor demonstrates significantly lower mean absolute ADC values compared with noncancerous tissue regardless of zonal origin (60). ADC measurements also correlate with tumor Gleason score, with clinically significant tumors demonstrating lower ADC values (61,62). In the context of focal therapy for primary prostate cancer, however, caution is needed when interpreting absolute ADC values, as posttreatment fibrosis also demonstrates low ADC values. To distinguish between fibrosis and cancer recurrence, the ADC map must be read in conjunction with high-*b*-value DWI, which is sensitive to changes in tissue microstructure. Cancer recurrence will demonstrate concomitant hyperintense signal at DWI, while fibrosis will demonstrate hypointense signal. A study by Velaga et al (63) investigating the performance of mpMRI in a cohort 1 year after focal cryotherapy showed that ADC values, when interpreted in conjunction with high-*b*-value DWI, were lower in clinically significant prostate cancer compared with benign histology and clinically insignificant prostate cancer.

### Pitfall: Periprostatic Fibrosis and Scarring May Cause Changes in the Prostatic Capsule and Impede Assessment of Extraprostatic Extension in Recurrent Tumors

Prostate cancer with extraprostatic extension reduces overall and cancer-specific survival. In the treatment-naive prostate, signs of extraprostatic extension include bulge and irregular prostate contour, capsular disruption, periprostatic fat infiltration, and rectoprostatic angle obliteration (64). Following focal cryoablation, fibrosis and capsular retraction may obliterate the capsule, rendering accurate assessment of extraprostatic extension in recurrent tumors extremely challenging.

## Medium- and Long-term Surveillance After Cryotherapy

The 2024 TARGET international consensus recommends further surveillance MRI 12 months after the first posttreatment MRI examination if the patient had negative findings at the first MRI examination and a normal PSA level, regardless of whether the patient underwent biopsy after the first MRI examination, even if that biopsy’s findings were negative. However, there was no consensus on how many years patients should undergo protocol MRI surveillance for, although this duration should be dependent on the patient’s clinicopathologic disease characteristics (41).

Similarly, with the 2020 international consensus, no consensus was reached as to whether further imaging is mandatory beyond the 6- and 18-month postprocedural mpMRI studies if test results were negative. Further imaging was recommended, however, in the event of new triggering factors such as new clinical suspicion, young age, genetic predisposition, and rise in PSA level, or as according to prevailing local institutional active surveillance protocols (11).

The 2020 international consensus also recommends systematic 12-core transrectal US-guided biopsy to evaluate the untreated area, together with a targeted biopsy of the treated area, performed 6–12 months after focal therapy (11). On the other hand, Wyscok and colleagues (12) observed low 2-year in-field and out-of-field clinically significant prostate cancer detection rates of 3% and 15%, respectively (12). Hence, they no longer mandate surveillance biopsy at 6 months but instead offer biopsy to patients with progressively rising PSA levels or with 2-year mpMR images with suspicious features. They advocate that early 6-month postprocedural biopsy should not be mandatory but should rather reflect biopsy outcomes at a local level.

### Pearl: Following Focal Therapy, mpMRI Continues to Be a Crucial Component of Medium- and Long-term Active Surveillance

**Figure 6:**
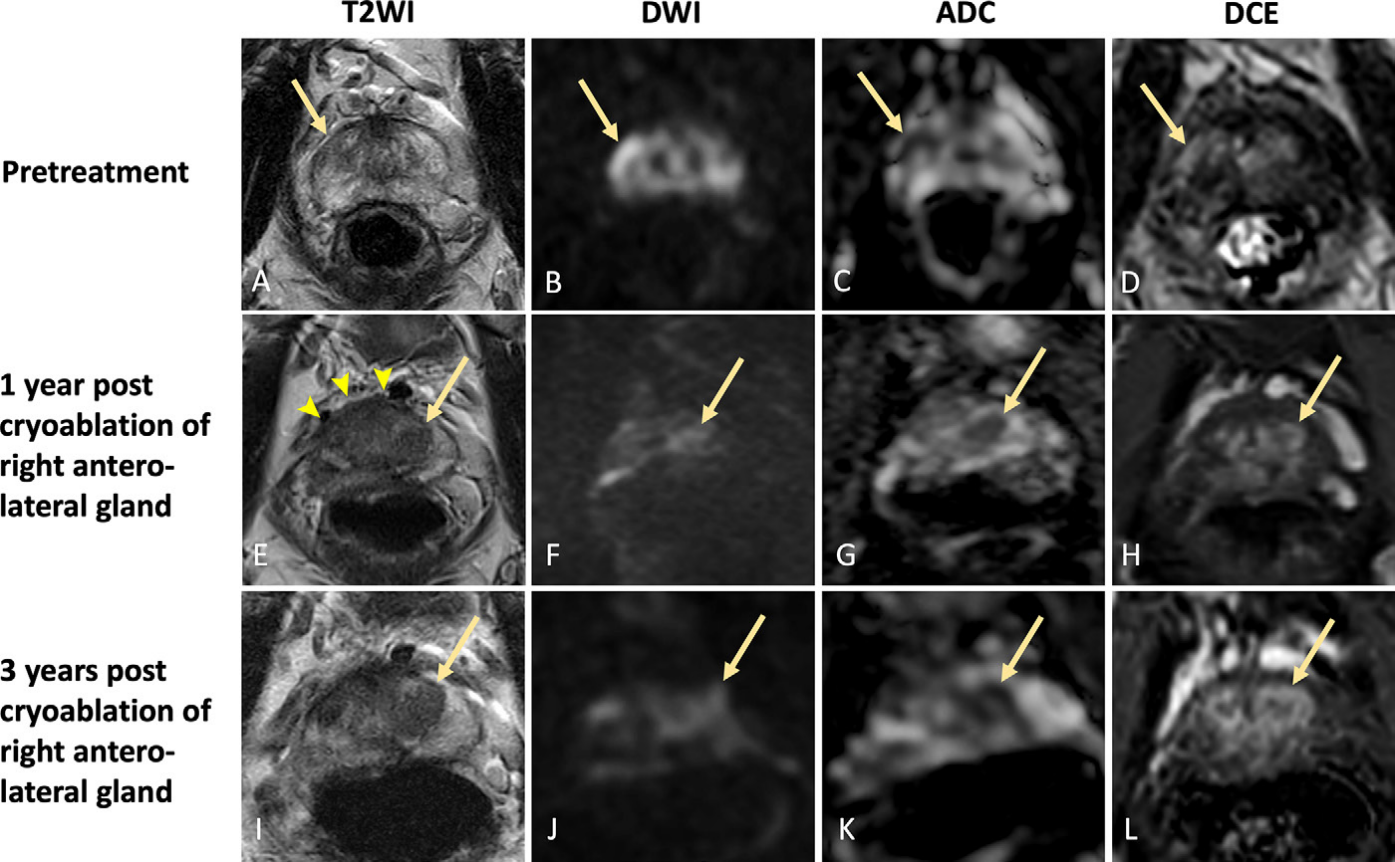
Images in a 62-year-old male patient with prostate cancer. Case illustrates challenges of targeted biopsy after focal therapy with out-of-field recurrence despite prior negative biopsy findings. **(A–D)** Images from pretreatment MRI. **(A)** Axial T2-weighted image, **(B)** high-*b*-value DW image, **(C)** ADC map, and **(D)** DCE image show T2-weighted hypointense lesion in the subcapsular right anterior peripheral zone midgland with restricted diffusion and enhancement (arrow). Lesion was assigned PI-RADS category 4. Patient underwent focal cryoablation of the right anterolateral apex and midgland. **(E–H)** MR images 1 year after focal cryoablation. **(E)** Axial T2-weighted image shows hypointense scarring in the right anterior peripheral zone with volume loss and capsular retraction (arrowheads). No focus of restricted diffusion or enhancement is seen at the ablation zone to suggest residual tumor. However, there is interval development of a PI-RADS 3 lesion in the left anterior transition zone (arrow) with **(E)** T2-weighted hypointense signal, **(F)** moderately hyperintense signal on DW image, **(G)** hypointense signal on ADC map, and **(H)** enhancement. Findings from 1-year postablation surveillance targeted biopsy at the left anterior transition zone were negative. **(I–L)** MR images 3 years after focal cryoablation. **(I)** Axial T2-weighted image, **(J)** high-*b*-value DW image, **(K)** ADC map, and **(L)** DCE image show that the lesion in the left anterior transition zone (arrow) has increased in size with persistent restricted diffusion, now PI-RADS 4. Biopsy was performed and histopathology revealed Gleason grade 4 + 3 out-of-field recurrence. Earlier negative biopsy findings may have been due to sampling error because of postablation scarring and fibrosis. ADC = apparent diffusion coefficient, DCE = dynamic contrast-enhanced, DWI = diffusion-weighted imaging, PI-RADS = Prostate Imaging Reporting and Data System, T2WI = T2-weighted imaging.

In our experience, mpMRI is a good complement to periodic histologic sampling. Targeted biopsy after focal therapy is often challenging, as scarring and fibrosis may give rise to sampling error (Fig 6). In such cases, close imaging follow-up is helpful to detect and monitor recurrent tumor.

**Figure 7:**
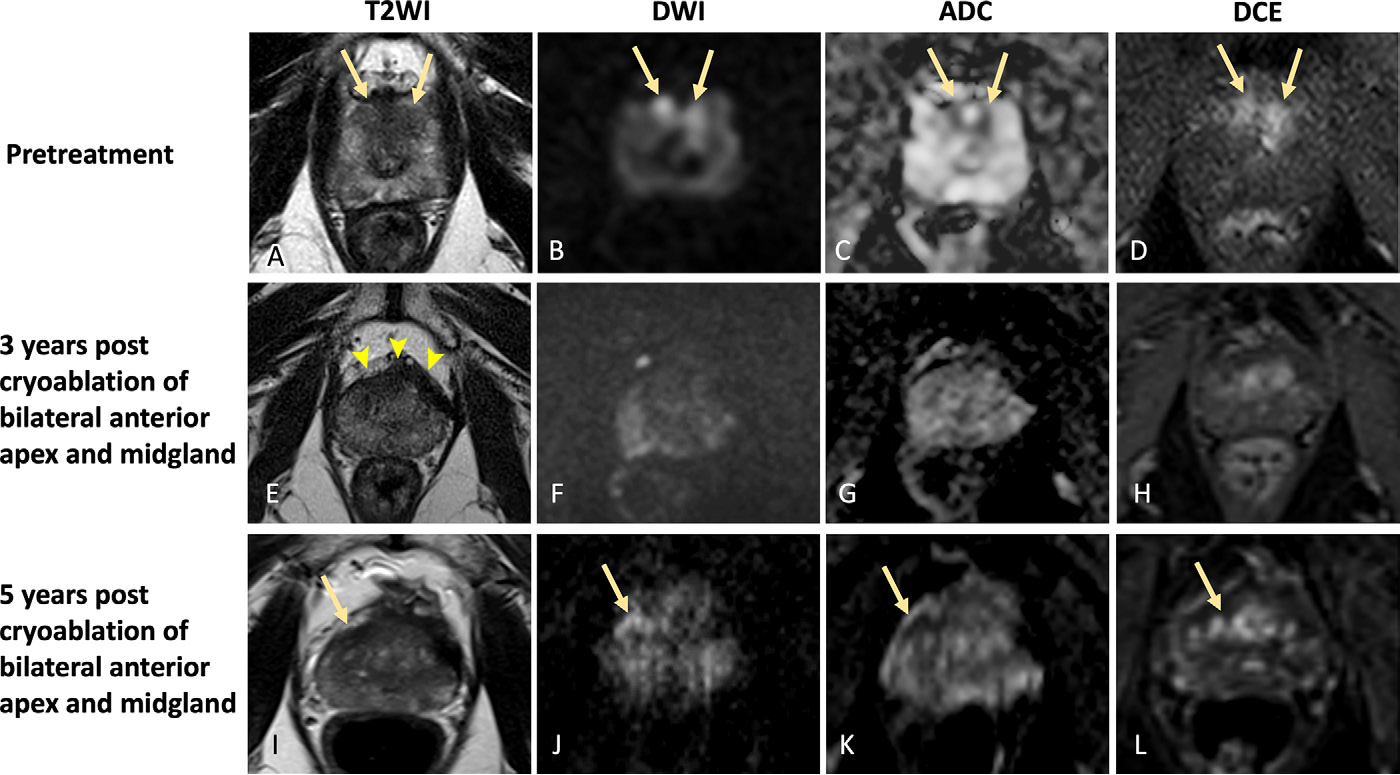
Images in a 62-year-old male patient with prostate cancer. Case illustrates in-field recurrence apparent at 5-year postcryoablation surveillance mpMRI despite prior negative findings at mpMRI and biopsy of the ablation site. **(A–D)** Images from pretreatment MRI. **(A)** Axial T2-weighted image, **(B)** high-*b*-value DW image, **(C)** ADC map, and **(D)** DCE image show hypointense lesions in the right and left anterior peripheral zone apex with restricted diffusion and enhancement (arrows). Lesions were assigned PI-RADS category 4. Patient underwent focal cryoablation of bilateral anterior apex and midgland. Findings from 1-year postablation MRI (not shown) were negative for residual or recurrent tumor. Systematic biopsy findings 1 year after focal therapy were also negative. **(E–H)** MR images 3 years after focal cryoablation. **(E)** Axial T2-weighted image shows hypointense scarring in bilateral anterior peripheral zone with volume loss and capsular retraction (arrowheads). No suspicious focus is seen on **(F)** high-*b*-value DW image, **(G)** ADC map, and **(H)** DCE image to suggest residual or recurrent tumor. **(I–L)** MR images 5 years after focal cryoablation. A lesion is now seen in the right anterior peripheral zone apex (arrow). **(I)** On T2-weighted image, the lesion is obscured by hypointense scarring, but it demonstrates **(J)** hyperintense signal on high-*b*-value DW image, **(K)** hypointense signal on ADC map, and **(L)** enhancement on DCE image. Post–focal therapy MRI finding is PI-FAB 3/TARGET 5. Histopathology revealed Gleason grade 3 + 4 in-field recurrence. ADC = apparent diffusion coefficient, DCE = dynamic contrast-enhanced, DWI = diffusion-weighted imaging, PI-FAB = Prostate Imaging after Focal Ablation, PI-RADS = Prostate Imaging Reporting and Data System, T2WI = T2-weighted imaging, TARGET = Transatlantic Recommendations for Prostate Gland Evaluation with Magnetic Resonance Imaging After Focal Therapy.

In our institution, patients undergo routine surveillance mpMRI at 1, 3, and 5 years following cryoablation. From our experience, in-field recurrence may become apparent at 3- and 5-year surveillance mpMRI despite earlier negative mpMRI findings and negative biopsy findings at the ablation site (Fig 7). With longer-term surveillance, posttreatment changes have regressed and stabilized, while small tumor foci not detected at prior MRI or systematic biopsy have had time to progress and manifest.

While focal therapy has shown promising short- to medium-term outcomes, there are few data regarding long-term efficacy and oncologic control. Long-term studies are required for urologists and radiologists alike to better understand the role of focal therapy in management of prostate cancer.

## Management of In-field Persistence and Out-of-field Tumors Following Focal Therapy

Besides initial treatment, discussion has also revolved around management options of biopsy-proven tumor recurrence. For clinically significant in-field recurrence of prostate cancer, salvage focal therapy or whole-gland treatment such as radical prostatectomy or radiation therapy may be considered, depending on clinical judgment and expectation of success and patient preferences (11). However, in-field and out-of-field recurrence of clinically insignificant prostate cancer may not warrant active treatment, and active surveillance with serum PSA testing and mpMRI may suffice in such cases.

For out-of-field clinically significant prostate cancer, these may represent MRI-occult lesions at initial evaluation or de novo lesions that became clinically apparent or progression of known tumors at surveillance imaging. These patients may be offered salvage focal therapy, but challenges in targeting MRI-occult lesions may shift these patients toward whole-gland approaches (11). One advantage of focal therapy is the ability to repeat the procedure in cases of treatment failure, particularly if the reason for initial failure can be identified and surmounted (11).

## Conclusion

While awaiting long-term data, focal therapy is a promising and increasingly popular alternative for the treatment of primary prostate cancer. Radiologists play a crucial role in pretreatment selection and posttreatment surveillance of patients. It is important for radiologists to be cognizant of expected post–focal treatment changes as well as pitfalls in the detection of residual and recurrent tumor with mpMRI. Multidisciplinary discussion between radiologists, urologists, and pathologists is vital to improve patient care and optimize oncologic outcomes in this highly challenging and exciting field.
